# Supplementary material for: An open-source framework for large-scale, flexible evaluation of biomedical text mining systems
Source: J Biomed Discov Collab. 2008 Jan 29;3:1. doi: 10.1186/1747-5333-3-1 (PMC2276192; doi:10.1186/1747-5333-3-1)
Supplement: Additional file 1 — Evaluation platform source code. The additional file contains the software evaluation framework discussed in this paper. The most current release can be downloaded from SourceForge.net [1]. See the accompanying README file for instructions on installation and use. Also included in the distribution is a collection of UIMA wrappers for some commonly used BioNLP tools and annotated corpora. [file 1747-5333-3-1-S1.zip › bionlp_uima_repository-1.0.1/docs/api/src-html/edu/uchsc/ccp/uima/ae/util/converter/AnnotatorOverrider_AE.html]

```
001    package edu.uchsc.ccp.uima.ae.util.converter;
002    
003    import java.util.Iterator;
004    
005    import org.apache.uima.analysis_engine.ResultSpecification;
006    import org.apache.uima.analysis_engine.annotator.AnnotatorConfigurationException;
007    import org.apache.uima.analysis_engine.annotator.AnnotatorContext;
008    import org.apache.uima.analysis_engine.annotator.AnnotatorContextException;
009    import org.apache.uima.analysis_engine.annotator.AnnotatorInitializationException;
010    import org.apache.uima.analysis_engine.annotator.AnnotatorProcessException;
011    import org.apache.uima.analysis_engine.annotator.JTextAnnotator_ImplBase;
012    import org.apache.uima.jcas.JCas;
013    
014    import edu.uchsc.ccp.uima.annotation.CCPAnnotator;
015    import edu.uchsc.ccp.uima.annotation.CCPTextAnnotation;
016    
017    public class AnnotatorOverrider_AE extends JTextAnnotator_ImplBase {
018    
019        public static final String PARAM_ANNOTATOR_ID = "AnnotatorID";
020        public static final String PARAM_FIRST_NAME = "FirstName";
021        public static final String PARAM_LAST_NAME = "LastName";
022        public static final String PARAM_AFFILIATION = "Affiliation";
023        
024        private int annotatorID = -1;
025        private String firstName = null;
026        private String lastName = null;
027        private String affiliation = null;
028        
029        @Override
030        public void initialize(AnnotatorContext ac) throws AnnotatorInitializationException, AnnotatorConfigurationException {
031            /* read in input parameters */
032            try {
033                annotatorID = ((Integer) ac.getConfigParameterValue(PARAM_ANNOTATOR_ID)).intValue();
034                firstName = (String) ac.getConfigParameterValue(PARAM_FIRST_NAME);
035                lastName = (String) ac.getConfigParameterValue(PARAM_LAST_NAME);
036                affiliation = (String) ac.getConfigParameterValue(PARAM_AFFILIATION);
037            } catch (AnnotatorContextException e) {
038                e.printStackTrace();
039            }
040            super.initialize(ac);
041        }
042        
043        
044        /**
045         * cycle through all annotations and set the annotation set
046         */
047        public void process(JCas jcas, ResultSpecification arg1) throws AnnotatorProcessException {
048    
049            /* create an annotation set list */
050            CCPAnnotator ccpAnnotator = new CCPAnnotator(jcas);
051            ccpAnnotator.setAnnotatorID(annotatorID);
052            ccpAnnotator.setAffiliation(affiliation);
053            ccpAnnotator.setFirstName(firstName);
054            ccpAnnotator.setLastName(lastName);
055            
056            
057            Iterator annotIter = jcas.getJFSIndexRepository().getAnnotationIndex(CCPTextAnnotation.type).iterator();
058    
059            while (annotIter.hasNext()) {
060                Object possibleAnnot = annotIter.next();
061                if (possibleAnnot instanceof CCPTextAnnotation) {
062                    CCPTextAnnotation ccpTA = (CCPTextAnnotation) possibleAnnot;
063                    ccpTA.setAnnotator(ccpAnnotator);
064                } else {
065                    System.err.println("WARNING -- AnnotatorOverrider_AE: CCPTextAnnotation expected but instead got "
066                            + possibleAnnot.getClass().getName());
067                }
068            }
069    
070        }
071    
072        
073    
074    }
```
